# Interleukin-10 and soluble tumor necrosis factor receptor II are potential biomarkers of *Plasmodium falciparum* infections in pregnant women: a case-control study from Nanoro, Burkina Faso

**DOI:** 10.1186/s40364-017-0114-7

**Published:** 2017-12-13

**Authors:** E. Ruizendaal, H. D. F. H. Schallig, J. Bradley, M. Traore-Coulibaly, P. Lompo, U. d’Alessandro, S. Scott, F. Njie, S. H. Zango, O. Sawadogo, M. D. de Jong, H. Tinto, P. F. Mens

**Affiliations:** 1 Department of Medical Microbiology, Academic Medical Centre, Amsterdam, The Netherlands; 2 Medical Research Council (MRC) Tropical Epidemiology Group, London School of Hygiene and Tropical Medicine, London, UK; 3 Institut de Recherche en Sciences de la Santé- Unité de Recherche Clinique de Nanoro, (IRSS-URCN), Nanoro, Burkina Faso; 4 Disease Control and Elimination, Medical Research Council Unit, Fajara, Gambia; 5 Department of Disease Control, Faculty of Infectious and Tropical Diseases, London School of Hygiene and Tropical Medicine, London, UK; 6 Department of Infectious Disease Epidemiology, London School of Hygiene and Tropical Medicine, London, UK

**Keywords:** Malaria, *Plasmodium falciparum*, Pregnancy, Placenta, Biomarkers

## Abstract

**Background:**

Diagnosis of malaria in pregnancy is problematic due to the low sensitivity of conventional diagnostic tests (rapid diagnostic test and microscopy), which is exacerbated due to low peripheral parasite densities, and lack of clinical symptoms. In this study, six potential biomarkers to support malaria diagnosis in pregnancy were evaluated.

**Methods:**

Blood samples were collected from pregnant women at antenatal clinic visits and at delivery. Microscopy and real-time PCR were performed for malaria diagnosis and biomarker analyses were performed by ELISA (interleukin 10, IL-10; tumor necrosis factor-α, TNF-α; soluble tumor necrosis factor receptor II, sTNF-RII; soluble fms-like tyrosine kinase 1, sFlt-1; leptin and apolipoprotein B, Apo-B). A placental biopsy was collected at delivery to determine placental malaria.

**Results:**

IL-10 and sTNF-RII were significantly higher at all time-points in malaria-infected women (*p* < 0.001). Both markers were also positively associated with parasite density (*p* < 0.001 and *p* = 0.003 for IL-10 and sTNF-RII respectively). IL-10 levels at delivery, but not during pregnancy, were negatively associated with birth weight. A prediction model was created using IL-10 and sTNF-RII cut-off points. For primigravidae the model had a sensitivity of 88.9% (95%CI 45.7–98.7%) and specificity of 83.3% (95% CI 57.1–94.9%) for diagnosing malaria during pregnancy. For secundi- and multigravidae the sensitivity (81.8% and 56.5% respectively) was lower, while specificity (100.0% and 94.3% respectively) was relatively high. Sub-microscopic infections were detected in 2 out of 3 secundi- and 5 out of 12 multigravidae.

**Conclusions:**

The combination of biomarkers IL-10 and sTNF-RII have the potential to support malaria diagnosis in pregnancy. Additional markers may be needed to increase sensitivity and specificity, this is of particular importance in populations with sub-microscopic infections or in whom other inflammatory diseases are prevalent.

**Electronic supplementary material:**

The online version of this article (10.1186/s40364-017-0114-7) contains supplementary material, which is available to authorized users.

## Background

In sub-Saharan Africa, an estimated 28 million pregnant women are at risk of contracting malaria [1]. Malaria during pregnancy caused by *Plasmodium falciparum* can have severe health consequences, such as maternal anemia, low birth weight, preterm birth, or even abortions and stillbirth [2, 3]. In Africa, up to 100.000 infant deaths per year are related to low birth weight due to malaria in pregnancy [4]. Normally, the adult population in malaria endemic areas has acquired (partial) immunity against the parasite due to repeated exposure throughout their lives, suppressing clinical symptoms [5, 6]. This immunity is for a great part directed against surface antigens of the *P. falciparum*-infected erythrocyte. However, during malaria in pregnancy a unique surface antigen on *P. falciparum-*infected erythrocytes is expressed, against which immunity is lacking [7]. This surface antigen is the variant surface antigen 2-chrondroitin sulphate A (VAR2CSA) antigen, a member of the *P. falciparum* erythrocyte membrane protein 1 (PfEMP1) family of surface antigens. With this pregnancy specific antigen, *P. falciparum* parasites can sequester in the placenta by binding to chondroitin sulphate A on syncytiotrophoblasts, thereby evading splenic clearance [8, 9]. This condition is known as placental malaria (PM) and can be further characterized by subsequent attraction of mononuclear cells into the intervillous space, changes in pro- and anti-inflammatory cytokine levels, hemozoin deposition in fibrin and impaired angiogenesis [10–14]. The burden of PM and associated LBW is most severe in primigravidae, while it wanes in multigravid women. This is because antibodies against VAR2CSA antigens are particularly low or absent in primigravidae who are exposed to the antigen for the first time, while women generally acquire VAR2CSA antibodies during successive pregnancies [7, 11, 15].

Currently, one of the main methods to prevent malaria in pregnancy and its related morbidity is intermittent preventive treatment with sulfadoxine-pyrimethamine (IPTp-SP), which consists of systematically administering SP at antenatal care (ANC) visits from second trimester onwards, regardless of malaria infection status [16]. Although this strategy is currently effective in preventing malaria-related low birth weight [17], the increasing resistance against SP and the declining overall malaria prevalence may warrant alternative approaches such as intermittent screening and treatment [1, 18]. However, due to low parasite densities, standard diagnostic methods like microscopy and rapid diagnostic tests (RDTs) are suboptimal [19, 20]. Molecular diagnostic techniques such as (real-time) polymerase chain reaction (PCR), are usually of limited use in endemic settings because of technical, infra-structural and financial requirements. Therefore, there is the need for alternative diagnostic approaches to reliably diagnose *P. falciparum* infections during pregnancy.

In the current diagnostic landscape, there is increasing interest in identifying and using host biomarkers to diagnose infection; for malaria in pregnancy, several potential biomarkers have been suggested (systematically reviewed by Ruizendaal et al.) [12]. Based on this review, a combination of markers of inflammation, angiogenesis and lipid metabolism was selected for further evaluation in the current study. The included biomarkers were: inflammatory markers interleukin 10 (IL-10), tumor necrosis factor-α (TNF-α) and soluble tumor necrosis factor receptor II (sTNF-RII); angiogenesis marker ‘soluble fms-like tyrosine kinase 1’ (sFlt-1); and markers of lipid metabolism leptin and apolipoprotein B (Apo-B). The levels of these potential biomarkers were studied in malaria-infected and uninfected pregnant women living in Nanoro, Burkina Faso. This is a highly endemic area for malaria in which pregnant women are commonly infected with malaria, albeit frequently without clinical symptoms [21]. Women were followed-up during pregnancy until delivery. Malaria diagnosis and biomarker analysis was performed at three time-points: second trimester, third trimester and delivery.

## Methods

### Study area

The study was conducted in the Nanoro health centre catchment area, situated approximately 85 km North-West of Ouagadougou, the capital of Burkina Faso. Malaria is highly seasonal with intense transmission during the rainy months (June – October), with a peak towards the end of the rainy season.

### Study population, procedures and design

This study aimed to identify host biomarkers that can be used to support malaria diagnosis. The study was nested within a cluster-randomized controlled trial (COSMIC study, trial registration numbers ISRCTN372259296 Current Controlled Trials and NCT01941564 clinicaltrials.gov). Full details of the trial have been reported elsewhere [22]. In the cluster-randomized controlled trial 1800 women from 30 different villages were included, of which 160 women were included in the current case-control study. Pregnant women were enrolled at first ANC visit and followed-up until delivery. Women were enrolled in the study if they were resident in the area and did not report hypersensitivity to sulphonamides. Women known to be HIV infected were excluded.

At each ANC visit, women received standard antenatal care, including intermittent preventive treatment with sulfadoxine-pyrimethamine according to national guidelines and as advised by the World Health Organization. [16] Furthermore, demographic and clinical data were reported in a case record form and a blood slide and blood spots on Whatman 3MM or 1 MM filter paper (spots of at least 12 mm diameter) were collected from finger prick blood. At first ANC visit (usually second trimester), second or third ANC visit (third trimester) and at delivery a venous blood sample was collected in vacutainer tubes containing ethylenediaminetetraacetic acid (EDTA). At delivery, birth weight was registered and a placental biopsy for histological analysis was collected. Gestational age at delivery was assessed by the new Ballard score [23].

The current study was set up as a case-control study. Initial cases and controls were defined by 18S *P. falciparum* real-time PCR results from peripheral blood spots collected at delivery. By using a random number generator, 79 malaria positive (cases) and 81 malaria negative (controls) pregnant women were selected. Blood samples were analysed using commercial ELISA kits for the 6 selected biomarkers (IL-10, TNF-α, sTNF-RII, sFlt-1, leptin and Apo-B). For statistical analyses of biomarker results the case-control definition was abandoned, instead the following definitions of malaria infection were used: peripheral malaria infection (positive by real-time PCR or microscopy, see below for methods) or placental malaria infection (acute or chronic infection, see below for methods).

### DNA extraction and real-time PCR of filter paper blood spots

Blood spots on filter paper collected at ANC visits and delivery were air dried, sealed in bags with silica and transported to the central laboratory (Unité de Recherche Clinique de Nanoro, URCN) where they were stored at ambient temperature until shipment to the Netherlands (Academic Medical Centre, Amsterdam) for molecular analyses. For each woman at each of the three time-points one blood spot per collected filter paper was punched using Acu-punch skin biopsy punchers (acuderm® inc, Florida, USA) and transferred to a 5 mL polystyrene tube. DNA extraction was performed using the Nuclisens EasyMag (bioMérieux, Marcy-l’Étoile, France) system as previously described [24]. Samples of extracted DNA were stored at −20 °C. *P. falciparum* DNA was detected by real-time PCR as previously described with some minor adjustments in primer concentrations and probe sequence [24–27]. In each PCR run a dilution series of *P. falciparum* FCR3 culture was included (10^4^ parasites/μL – 1 parasites/μL), as well as positive (FCR3 spiked EDTA blood from healthy donors, Dutch blood bank) and negative extraction controls (EDTA blood from healthy donors, Dutch blood bank).

### Microscopy slides

Blood slides collected at ANC visits and delivery were Giemsa stained (3%) for 45–60 min. Slides were read independently by two microscopists. Parasites were counted against 200 leukocytes, or 500 leukocytes if the count was less than 10 parasites/200 leukocytes. Slides were considered negative if no parasite was detected in 100 high power fields. Any discrepancies in the two readings were resolved by a third independent reader.

### Placental histology

Placental biopsies of ±1 cm^3^ were collected from the maternal side of the placenta and preserved in 10% neutral buffered formalin. The tissue was subsequently embedded in paraffin wax and stored at 4 °C until shipment to the Medical Research Council The Gambia (MRCG) for further evaluation. Four millimeter thick paraffin embedded tissue sections were stained with hematoxylin-eosin after which they were read by a trained microscopist. Spot checks were done by a senior lab technician on a random sample of biopsies. Histology results were categorized into: acute infection (parasites present, but no pigment), chronic infection (both parasites and pigment present), past infection (only pigment present) and no infection (no parasites, no pigment) [28].

### Biomarker ELISA

Venous blood samples collected at ANC visits and at delivery were stored and transferred in cooling boxes to the central laboratory in Nanoro within one day. The exact time and date of blood collection as well as the time and date of further processing of the sample in the laboratory were documented to enable estimation of storage and transfer time. Blood samples were centrifuged at 1000 g for 15 min after which the plasma was aliquoted in 1.5 mL cryotubes and stored at −80 °C. Plasma samples were shipped to the Netherlands (Academic Medical Centre, Amsterdam) on dry ice for further analyses. Commercially available quantikine ELISA kits for human samples were used according to the manufacturer’s instructions to test the following biomarkers: IL-10, TNF-α, sTNF-RII, sFlt-1, leptin and Apolipoprotein-B (R&D Systems, Minneapolis, USA). The sensitivities of the quantikine ELISA kits for the respective biomarkers were 3.9 pg/mL, 5.5 pg/mL, 2.3 pg/mL, 13.3 pg/mL, 7.8 pg/mL and 9.97 ng/mL. Measurements below the detection limit were given half the concentration of the detection limit.

### Statistical analyses

Data were analysed using Stata 14.1. Differences in baseline characteristics such as age, haemoglobin, gravidity, infection status at enrolment and delivery and (low) birth weight were analysed by linear or logistic regression with robust standard errors to account for clustering of women in villages. Regression analyses with robust standard errors were also used to associate the biomarkers with peripheral and placental malaria infection, (low) birth weight, parasite density and storage time. Sub-analyses were performed for trimester of sample collection and gravidity. A diagnostic model was created by selecting the biomarkers with clinically relevant and significant differences between malaria-infected and uninfected women. The dataset was randomly divided into a test and validation set. Based on the distributions of the test set in both malaria positive and malaria negative women, cut-off points were manually determined for the biomarkers of interest. Sensitivity and specificity for the different end scores in the validation set were calculated using logistic regression analyses with robust standard errors. Confidence intervals (95%) and *p* values are reported, unadjusted for multiple comparisons.

## Results

**Fig. 1:**
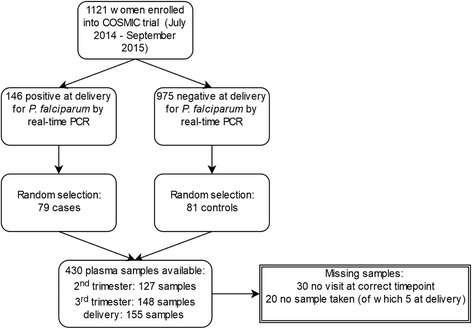
Flow chart of participants included in the biomarker analyses

For the randomly selected 79 cases and 81 controls, 430 plasma samples were available for biomarker analyses (Fig. 1). The Ballard score was missing for 22 participants (13.8%), while the available scores resulted in a wide unrealistic gestational age distribution with a range of 34 up to 47 weeks. Therefore, the trimester of sample collection was determined by considering samples collected at delivery as full term (*n* = 155), samples collected during 84 days prior to delivery as third trimester (*n* = 148) and those collected more than 84 days prior to delivery as second trimester samples (*n* = 127). The mean age was 25.4 ± 6.0 years and none of the participants showed signs of preeclampsia during follow-up. Hemoglobin levels at delivery were equal in cases and controls. There were slightly more primigravidae among cases (25.3%) than controls (19.8%), but the difference was not statistically significant (*p* = 0.371). There were no differences in infection rates at enrollment between cases and controls, both by real-time PCR (48.7 versus 46.9%, *p* = 0.799) and microscopy (30.4 versus 32.1%, *p* = 0.798). At delivery, only 43% of cases (34/79) detected by real-time PCR were also positive by microscopy. In contrast, most real-time PCR negative samples at delivery were also microscopy negative (only 2.5%, 2 out of 81, tested positive). Only 11.9% (8/67) of women with a peripheral infection detected by real-time PCR had a concurrent active placental infection, while 4.5% (3/67) of real-time PCR negative controls had an active placental infection (Table 1). The birthweight and the prevalence of low birth weight babies did not significantly differ between cases and controls. The median number of IPTp-SP doses was 3 (IQR 2–4), which did not significantly differ between cases and controls.

**Table 1:** Clinical characteristics of participants included in the biomarker analyses

	Total (*n* = 160)	Cases (*n* = 79)	Controls (*n* = 81)	*p* value
Age in years, mean (SD)	25.4 (± 6.0)	25.2 (± 6.2)	25.7 (± 5.8)	0.560
Preeclampsia	0	0	0	–
Hemoglobin at delivery in g/dL mean (SD)	12.0 (1.5)	11.9 (1.6)	12.1 (1.4)	0.527
Gravidity				
Primigravidae % (*n*)	22.5 (36)	25.3 (20)	19.8 (16)	0.371
Secundigravidae % (*n*)	17.5 (28)	16.5 (13)	18.5 (15)	0.784
Multigravidae % (*n*)	60 (96)	58.2 (46)	61.7 (50)	0.595
Infected at first visit				
By microscopy % (*n*)	31.3 (50/160)	30.4 (24/79)	32.1 (26/81)	0.798
By real-time PCR % (*n*)	47.8 (76/159)	48.7 (38/78)	46.9 (38/81)	0.799
Infected at delivery				
By microscopy, % (*n*)	22.5 (36/160)	43.0 (34/79)	2.5 (2/81)	<0.001
By placental biopsy, % (*n*)	8.2 (11/134)	11.9 (8/67)	4.5 (3/67)	0.165
Birthweight in kg, mean (SD)	3.01 (0.57)	2.94 (0.62)	3.07 (0.52)	0.130
Low birth weight < 2500 g, % (*n*)	9.5 (15/158)	7.7 (6/78)	11.3 (9/80)	0.410
IPTp-SP doses, median (IQR)	3 (2–4)	3 (2–4)	3 (2–3)	0.627

### Stability of biomarkers in EDTA blood

The influence of the storage and transport time of EDTA samples to the central laboratory on the markers was assessed (Additional file 1: Table S1). None of the markers were significantly affected by time, except for sFlt-1 which slowly increased over time (*p* = 0.001). In multivariate analyses including storage and transport time, none of the associations between biomarkers and peripheral or placental malaria infection were affected.

### Biomarker levels in women with and without peripheral malaria infections

**Fig. 2:**
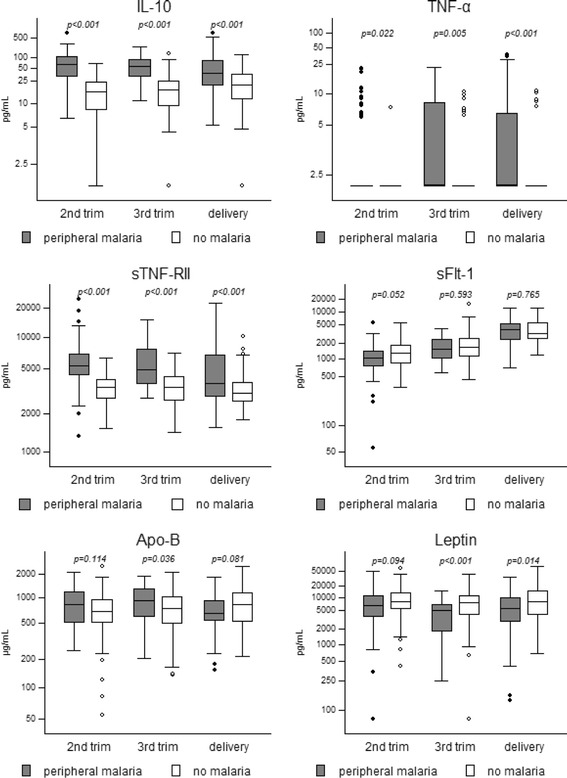
Biomarker levels in women with peripheral malaria infections versus women without peripheral infection. Box plots presenting the median (horizontal lines), IQR (boxes), adjacent values (whiskers) and outliers (dots). *p* values from regression analyses with robust variance estimators are given; trim = trimester; numbers with peripheral malaria (real-time PCR and/ or microscopy positive)/ total tested: ‘2nd trimester’ 65/62, ‘3rd trimester’ 32/116, ‘delivery’ 79/76 for IL-10, TNF-α, Leptin and Apo-B; ‘2nd trimester’ 64/59, ‘3rd trimester’ 29/112, ‘delivery’ 66/67 for sFlt-1; ‘2nd trimester’ 50/59, ‘3rd trimester’ 29/115, ‘delivery’ 68/75 for sTNF-RII

At each time point (second trimester, third trimester and delivery) the levels of the biomarkers were compared between women with and without peripheral malaria infection (Fig. 2). Peripheral infection was defined as being real-time PCR and/or microscopy positive for *P. falciparum.* For nine biomarker measurements during pregnancy the real-time PCR data was missing, and one microscopy result was missing. For sFlt-1 and sTNF-RII there were 33 (7.7%) and 34 (7.9%) samples respectively that fell outside the range of the standard curve. Repeated measurements were not conclusive as the adjusted mean difference was >10%. Therefore these measurements were excluded from the initial analyses, but included for sensitivity analyses (see below).

Both inflammatory markers IL-10 and sTNF-RII were significantly higher in infected than in uninfected women at all time-points (*p* < 0.001) (Fig. 2). Although TNF-α also showed significant higher levels in malaria infected women, many samples were below the TNF-α assay detection limit (127/176 samples with peripheral infection and 238/254 uninfected samples). The remaining biomarkers did not show consistent differences between malaria infected and uninfected women during pregnancy, except for leptin, but in second trimester this difference was not significant (*p* = 0.094). Multivariate analyses, including age and gravidity, did not substantially change the associations between any of the markers and malaria infection. Sensitivity analyses including the primary or repeated measurements of the excluded sTNF-RII and sFlt-1 samples, did not result in different conclusions, except that *p* values for the decrease in sFlt-1 levels in second trimester were significant (*p* = 0.012, *p* = 0.017 and *p* = 0.029 for primary and repeated measurements respectively).

### Sub-microscopic peripheral malaria infections and biomarker levels

**Fig. 3:**
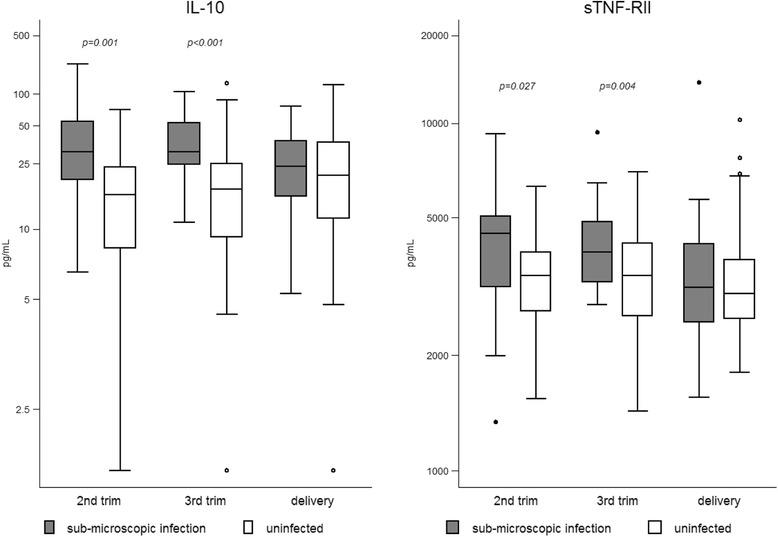
Biomarker levels in women with sub-microscopic *P. falciparum* infections versus uninfected women. Box plots presenting the median (horizontal lines), IQR (boxes), adjacent values (whiskers) and outliers (dots). *p* values from regression analyses with robust variance estimators are given; numbers with sub-microscopic infection/ uninfected: ‘2nd trimester’ 24/62, ‘3rd trimester’ 14/116, ‘delivery’ 44/76 for IL-10,; ‘2nd trimester’ 20/59, ‘3rd trimester’ 13/115, ‘delivery’ 40/75 for sTNF-RII

Sub-analyses were performed for differences in biomarker concentrations between women with sub-microscopic peripheral infections compared with uninfected women. Only IL-10 and sTNF-RII remained significantly increased in second and third trimester in women with sub-microscopic infections compared with uninfected women. There was no longer a difference in these cytokine levels between infected and uninfected women at delivery (Fig. 3).

### Biomarker levels in women with and without placental malaria infections

**Fig. 4:**
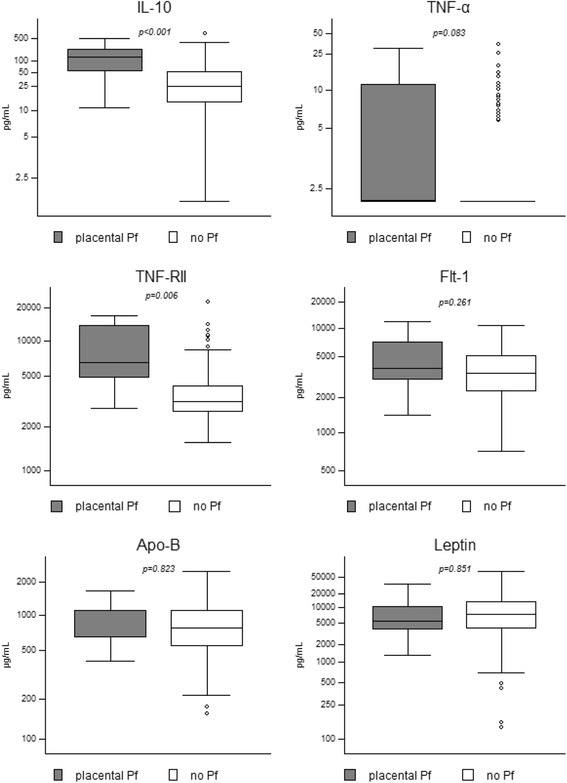
Biomarker levels between women with *P. falciparum* infected placentas versus women with uninfected placentas at time of delivery. Box plots presenting the median (horizontal lines), IQR (boxes), adjacent values (whiskers) and outliers (dots). *p* values from regression analyses with robust variance estimators are given; trim = trimester; Samples from women with placentas with parasitized erythrocytes (acute/chronic, *n* = 11) were compared with samples from women with placentas without parasitized erythrocytes (past/no infection, *n* = 119). For sTNF-RII these numbers were *n* = 9 for acute/chronic and *n* = 109 for past/no infection and for sFlt-1 *n* = 9 for acute/chronic and *n* = 101 for past/no infection. Pf = *P. falciparum* infection

IL-10 and sTNF-RII were significantly higher in women with an active placental infection (both acute and chronic) than in those without (*p* values <0.001 and 0.006 respectively) (Fig. 4). These two markers were also significantly increased at time of delivery if ‘any malaria infection’ (both placental and/or peripheral) was compared with no malaria infection (*p* < 0.001). Multivariate analyses, including age and gravidity, did not change the results.

Regression analyses were also performed for biomarker levels during pregnancy compared with a chronic malaria infection (active chronic or past chronic) at delivery. Increased IL-10, TNF-α, sTNF-RII and Apo-B levels in second trimester were significantly associated with chronic PM infections at delivery (*p* = 0.001; *p* = 0.025; *p* < 0.001 and *p* = 0.001 respectively). In the third trimester, higher levels of IL-10 and sTNF-RII were associated with an increased risk of chronic PM infection at delivery (*p* = 0.002 for both).

The biomarker levels at delivery were compared for women with chorioamnionitis (*n* = 44) and without (*n* = 22), as determined by placental histology. None of the markers showed significant differences (Additional file 1: Table S2).

### Biomarker levels in comparison with parasite density

Regression analyses for the association between each of the biomarkers and parasite density were based on microscopy results (Table 2). IL-10, TNF-α, sTNF-RII and sFlt-1 were positively associated with parasite density (*p* < 0.001, *p* = 0.001, *p* = 0.003 and *p* = 0.008, respectively). Multivariate analyses adjusting for gravidity and age did not change these associations. Repeating the analysis using real-time PCR estimations of parasite densities (including sub-microscopic infections) produced similar results, except that sFlt-1 was no longer significantly associated with parasite density (*p* = 0.344) (data not shown).

**Table 2:** Association of parasite density with biomarker levels by regression analyses with robust variance estimators

Biomarker	*N*	Coeff.^a^	95% CI		*p*
IL-10	93	0.25	0.15	0.35	<0.001
TNF-α	93	0.17	0.08	0.27	0.001
sTNF-RII	73	0.14	0.05	0.23	0.003
sFlt-1	84	0.17	0.05	0.29	0.008
Apo-B	93	−0.02	−0.09	0.05	0.632
Leptin	93	−0.05	−0.22	0.11	0.516

Biomarkers and parasite density are on the log scale. Parasite density was based on microscopy results

*Coeff* coefficient

^a^One log increase in biomarker per one log increase of parasite density

### Biomarker levels in comparison with birth weight

Birth weights were similar in women with peripheral malaria compared to women without peripheral malaria at delivery (*p* = 0.184) (Table 1). Birth weights were also similar between women with and without a placental infection (both for active or chronic placental infection; *p* = 0.873 and *p* = 0.488 respectively). An inverse correlation was seen for IL-10 levels and birth weight (*p* = 0.006) at delivery, but not for IL-10 concentrations in second and third trimester (Table 3). A positive correlation for Apo-B and birth weight was seen at delivery (*p* = 0.027) and also in second and third trimester (*p* = 0.020 and *p* = 0.076 respectively). However, in multivariate analyses adjusting for age, gravidity and malaria infection no associations were observed for any of the biomarkers and birth weight. Also, none of the markers were significantly associated with low birth weight (<2500 g) at any time point. (Additional file 1: Table S3).

**Table 3:** Association of biomarker levels with birth weight by regression analyses with robust variance estimators

Biomarker	*N*	Coeff.^a^	95% CI		*p*
Delivery					
IL-10	149	−0.30	−0.58	−0.03	0.006
TNF-α	149	0.05	−0.12	0.23	0.543
sTNF-RII	137	−0.09	−0.23	0.05	0.208
sFlt-1	127	0.01	−0.23	0.26	0.905
Apo-B	149	0.16	0.02	0.30	0.027
Leptin	149	−0.10	−0.67	0.17	0.447
Third trimester					
IL-10	141	−0.02	−0.10	0.06	0.680
TNF-α	141	0.06	−0.08	0.19	0.393
sTNF-RII	137	0.08	−0.16	0.32	0.493
sFlt-1	134	−0.16	−0.31	−0.01	0.037
Apo-B	141	0.11	−0.01	0.23	0.076
Leptin	141	−0.04	−0.12	0.05	0.365
Second trimester					
IL-10	123	0.00	−0.05	0.05	0.999
TNF-α	123	0.15	−0.11	0.41	0.240
sTNF-RII	105	0.03	−0.10	0.16	0.611
sFlt-1	119	−0.13	−0.37	0.11	0.264
Apo-B	123	0.12	0.02	0.23	0.020
Leptin	123	0.02	−0.10	0.14	0.733

Biomarkers are on the log scale, twins and stillborn babies excluded

*Coeff* coefficient

^a^One kg increase per one log increase of biomarker. Univariate analyses

### Prediction model using IL-10 and sTNF-RII for malaria during pregnancy

Participants of the study were randomly assigned to a test set (*n* = 80) and a validation set (*n* = 80). Based on the distributions of the individual biomarkers in malaria positive versus negative women (peripheral diagnosis) in the test set (Additional file 2: Figure S1), a scoring model using IL-10 and sTNF-RII levels was developed for predicting a malaria infection during second and third trimester (Tables 4 and 5). Delivery samples were not included in these evaluations, as for preventing malaria-related morbidity there is no clinical use in identifying *P. falciparum* infections at delivery. For IL-10, a score of 0 was given for measurements <30 pg/mL, a score of 1 was given for measurements between 30 and 75 pg/mL and a score of 2 was given measurements over 75 pg/mL. For sTNF-RII a score of 0 or 1 was given to levels below or above the threshold of 3700 pg/mL respectively. By using real-time PCR and microscopy as reference test, a cut-off value of 2 points (<2 is negative, ≥2 is positive) for the model in the validation set resulted in a sensitivity of 88.9% (95% CI 45.7–98.7) and specificity of 83.3% (95% CI 57.1–94.9) in primigravidae; 81.8% (95% CI 48.8–95.50) and 100.0% (95% CI 71.5–100.0) for secundigravidae; and 56.5% (95% CI 41.7–70.2) and 94.3% (95% CI 86.3–97.8%) for multigravidae (Table 5).

**Table 4:** Prediction model for malaria during pregnancy based on IL-10 and sTNF-RII levels

Biomarker	Threshold (pg/mL)	Points
IL-10	<30	0
	30–75	1
	>75	2
sTNF-RII	<3700	0
	>3700	1
Maximum		3

**Table 5:** Sensitivity and specificity for model cut-off scores compared with peripheral malaria by real-time PCR or microscopy

	Sensitivity%	95% CI		Specificity%	95% CI	
Primigravidae						
Model >0	100	66.4	100	54.2	34.9	72.3
Model >1	88.9	45.7	98.7	83.3	57.1	94.9
Model >2	77.8	44.5	93.9	100	85.8	100
Secundigravidae						
Model >0	90.9	51.1	99.0	63.6	30.4	87.5
Model >1	81.8	48.8	95.5	100	71.5	100
Model >2	54.5	20.1	85.2	100	71.5	100
Multigravidae						
Model >0	82.6	60.2	93.7	56.6	44.7	67.8
Model >1	56.5	41.7	70.2	94.3	86.3	97.8
Model >2	17.3	7.4	35.7	98.1	89.3	99.7

Model > 0: score 0 = negative, score > 0 = positive; Model > 1: score 0/1 = negative, score > 1 = positive; Model > 2: score 0–2 = negative, score > 2 = positive

Performance of the model was also assessed for microscopy negative samples only, to verify whether the model could be of use for diagnosing sub-microscopic infections. However, in our validation set none of the primigravidae had a sub-microscopic infection, hence the biomarkers model was of no additional value in these cases. For secundigravidae 2 out of 3 (66.7%) sub-microscopic infections would be detected by the model, without any false positive results. For multigravidae 5 out of 12 (41.7%) women with sub-microscopic infections would be identified, while 3 out of 51 (5.9%) women would have a false positive test result.

## Discussion

Two inflammatory markers were useful as biomarkers for malaria in pregnancy: the cytokine IL-10 and soluble cytokine receptor sTNF-RII. Both markers were significantly higher throughout pregnancy and at delivery in malaria infected women (both for peripheral malaria and placental malaria) than in uninfected women, as was shown previously [29–33]. Furthermore, higher levels of IL-10 and sTNF-RII during pregnancy were associated with chronic placental malaria at delivery. The fact that IL-10 and sTNF-RII have been identified as potential biomarkers in other studies with different populations of pregnant women and different methodologies, [29–33] reinforces the notion that both markers are strongly associated with malaria infection. This is supported also by the association of both markers with parasite density in this study. Interestingly, recent research also suggests that sTNF-RII in urine can be used as a biomarker for malaria in pregnancy [34].

As a novelty to previous research, the discriminative properties of the two markers were translated into a model for the diagnosis of malaria during pregnancy, with a high sensitivity (88.9%, 95% CI 45.7–98.7) and fair specificity (83.3% (95% CI 57.1–94.9) for primigravidae. For secundi- and particularly multigravidae the model was less adequate in terms of sensitivity, possibly because IL-10 and sTNF-RII responses are negatively influenced by the immunity acquired during previous pregnancies and by lower parasite densities. As primigravidae are at highest risk of malaria infection and of malaria-related morbidity [35], the model is still clinically relevant.

A model using host biomarkers would be most likely of use as an addition to current malaria diagnostics. For example, given the limited sensitivity of RDT and field microscopy, the biomarkers model might assist in identifying women with malaria infections not detected by these tests. Therefore, the additional value of the model was assessed for sub-microscopic infections (RDTs were not performed) in the current study. Unfortunately, the number of sub-microscopic infections were relatively small and none were found in primigravidae in the validation set. In secundi- and multigravidae, it seems that the model results in few false positive diagnoses of malaria, yet, the sensitivity for detecting sub-microscopic infections can still be improved.

Initially, the idea to generate sufficient sensitivity and specificity of the biomarkers model was the inclusion of markers from different groups or pathways involved in PM, e.g. sFlt-1, leptin and Apo-B, and not solely (anti)inflammatory markers. As these other markers could not be included in the model, it is unclear whether the combination of IL-10 and sTNF-RII is sufficiently specific for diagnosing malaria. Both markers are involved in inflammatory processes and may increase during infectious or inflammatory diseases other than malaria. IL-10, produced by numerous immune cells, has both anti- and pro-inflammatory effects: it can inhibit antigen presentation and pro-inflammatory Th1 cytokine expression, yet it can also have stimulatory effects on B-cells and natural killer cells (reviewed in [36, 37]). It was already shown that pregnant women with influenza also present with increased concentrations of IL-10. In contrast, IL-10 levels were within normal range in women infected with the parasitic disease filariasis [38, 39]. For sTNF-RII, that has mainly an anti-inflammatory effect by preventing circulating TNF-α from activating its cell bound receptors [40], there is a lack of available data on its dynamics in pregnant women with other infectious diseases. However, it is unlikely that increased sTNF-RII levels are unique to malaria infections, as higher levels have been found in non-pregnant individuals with for example dengue infection [41]. Furthermore, increased sTNF-RII levels have been associated with preeclampsia [42]. The combination of IL-10 and sTNF-RII as proposed in the model should therefore be evaluated in larger cohorts and in populations with other inflammatory diseases to assess its specificity. Furthermore, it would be of value to study additional promising markers based on recent literature and a literature review [12], e.g. the angiopoietins [43], low and high density lipoproteins (LDL and HDL) [44], complement component 5a (C5a) [14], or interferon-γ induced protein 10 (IP-10) [29, 30]. Interestingly, a recent study in non-pregnant individuals revealed that the combination of haptoglobin with sTNF-RII or IL-10 could be used to discriminate between bacterial, viral or malaria infections [45].

This study has some limitations. Inherent to this type of explorative biomarker study is the risk of overfitting when creating a diagnostic or predictive model. Furthermore, there was no information on concurrent infectious diseases other than chorioamnionitis that may have influenced the level of biomarkers in the study population. As stated before, the model should be evaluated in other populations, preferably in a prospective manner. Another limitation is the uncertainty in estimations of the gestational age by considering all births full-term, which may have resulted in some samples being allocated to the wrong trimester. However, because for most biomarkers there were no large differences in the results of samples collected in second or third trimester, it was not considered to be of substantial impact to the results. Nevertheless, the uncertainty in gestational age estimations made it impossible to relate the biomarkers to outcomes such as preterm birth or small for gestational age. For future surveys, one of the recommendations would be to perform ultrasound imaging throughout pregnancy to have reliable estimates of gestational age and, more importantly, to enable more direct comparisons of fetal growth with malaria infections and biomarker levels.

## Conclusions

This study has shown that both IL-10 and sTNF-RII are biomarkers of peripheral and placental malaria infection. More importantly, a new approach of combining these biomarkers in a prediction model resulted in high sensitivity and specificity for detecting microscopically proven malaria infections in primigravidae. However, as confidence intervals were large and there is a risk of overfitting, the potential of this model should be confirmed in other studies. Furthermore, as a biomarkers model is probably of most value for sub-microscopic infections, an increase in sensitivity for detecting (sub-)microscopic infections should be pursued, while at the same time specificity might need improvement if other infectious diseases are prevalent. The current model can therefore serve as a step-up for future pursuits in creating a diagnostic model for malaria in pregnancy. Addition of one or two other markers to the model, such as haptoglobin, C5a, angiopoietins, HDL/LDL, or IP-10 should be considered. In this era of rapidly changing malaria epidemiology, rapid spread of drug resistance and the increased demand for reliable diagnostics for malaria in pregnancy, the field of biomarkers could play an extremely important role.

## Additional files

Additional file 1: Table S1. Regression analyses of the association between storage and transport time (in hours) of EDTA samples and each of the biomarkers. **Table S2**. Regression analyses of the association between chorioamnionitis infection and each of the biomarkers at delivery. **Table S3**. Association of biomarker levels with low birth weight by regression analyses with robust variance estimators. (PDF 468 kb)

Additional file 2: Figure S1. Distribution of IL-10 and sTNF-RII in malaria infected versus uninfected women during pregnancy. (PDF 36 kb)

## Abbreviations

ANC: Antenatal care
Apo-B: Apolipoprotein B
C5a: Complement component 5a
HDL: High density lipopoprotein
IL-10: Interleukin-10
IP-10: Interferon-γ induced protein 10
IPTp-SP: Intermittent preventive treatment with sulfadoxine-pyrimethamine
LBW: Low birth weight
LDL: Low density lipoprotein
PCR: Polymerase chain reaction
PfEMP1: *Plasmodium falciparum* erythrocyte membrane protein 1
PM: Placental malaria
RDT: Rapid diagnostic test
sFlt-1: soluble fms-like tyrosine kinase 1
sTNF-RII: soluble tumor necrosis factor receptor II
TNF-α: Tumor necrosis factor alpha
VAR2CSA: Variant surface antigen 2-chondroitin sulphate A
