# Supplementary material for: Interleukin-10 and soluble tumor necrosis factor receptor II are potential biomarkers of Plasmodium falciparum infections in pregnant women: a case-control study from Nanoro, Burkina Faso
Source: Biomark Res. 2017 Dec 13;5:34. doi: 10.1186/s40364-017-0114-7 (PMC5729512; doi:10.1186/s40364-017-0114-7)
Supplement: Supplementary file 1 — Regression analyses of the association between storage and transport time (in hours) of EDTA samples and each of the biomarkers. Table S2. Regression analyses of the association between chorioamnionitis infection and each of the biomarkers at delivery. Table S3. Association of biomarker levels with low birth weight by regression analyses with robust variance estimators. (PDF 468 kb) [file 40364_2017_114_MOESM1_ESM.pdf]

Supplemental Table 1. Regression analyses of the association between storage and transport time (in hours) of EDTA samples and each of the biomarkers.

Biomarkers are on the log scale; time in hours

Biomarker	*n*	Coefficient	95% CI		*p*
IL-10	427	0.006	-0.015	0.028	0.554
TNF-α	427	-0.010	-0.020	0.000	0.059
sTNF-RII	393	0.002	-0.008	0.013	0.652
sFlt-1	394	0.027	0.012	0.042	0.001
Apo-B	427	0.002	-0.008	0.013	0.639
Leptin	427	-0.014	-0.031	0.003	0.097

Supplemental Table 2. Regression analyses of the association between chorioamnionitis infection and each of the biomarkers at delivery.

Biomarkers are on the log scale; Coeff = coefficient; 2 of women with chorioamnionitis did not have biomarker samples at delivery

Biomarker	*n*	Coeff.	95% CI		*p*
IL-10	64	0.30	-0.15	0.75	0.177
TNF-α	64	0.20	-0.07	0.47	0.148
sTNF-RII	58	-0.17	-0.36	0.02	0.083
sFlt-1	55	-0.21	-0.59	0.18	0.277
Apo-B	64	0.02	-0.22	0.27	0.851
Leptin	64	0.13	-0.43	0.70	0.629

Supplemental Table 3. Association of biomarker levels with low birth weight by regression analyses with robust variance estimators

Biomarkers are on the log scale, twins and stillborn babies excluded. OR = odds ratio; All TNF-α values in women who were malaria positive and gave birth to a low birth weight baby were undetectable.

Biomarker	*N*	OR	95% CI		*p*
Delivery					
IL-10	149	0.96	0.64	1.44	0.849
TNF-α	149	0.45	0.11	1.93	0.283
sTNF-RII	137	0.70	0.25	1.93	0.490
sFlt-1	127	1.15	0.39	3.34	0.800
Apo-B	149	0.50	0.15	1.66	0.257
Leptin	149	1.48	0.77	2.87	0.240
Third trimester					
IL-10	141	1.20	0.56	2.59	0.638
TNF-α	141	0.67	0.13	3.52	0.632
sTNF-RII	137	0.51	0.06	4.24	0.530
sFlt-1	134	1.32	0.45	3.91	0.615
Apo-B	141	0.67	0.25	1.80	0.429
Leptin	141	1.80	0.67	4.81	0.241
Second trimester					
IL-10	123	1.24	0.54	2.84	0.607
TNF-α	-	-	-	-	-
sTNF-RII	105	1.00	0.27	3.70	0.999
sFlt-1	119	0.38	0.06	2.21	0.280
Apo-B	123	0.81	0.25	2.56	0.715
Leptin	123	0.82	0.49	1.38	0.459
