# Supplementary material for: Interleukin-10 and soluble tumor necrosis factor receptor II are potential biomarkers of Plasmodium falciparum infections in pregnant women: a case-control study from Nanoro, Burkina Faso
Source: Biomark Res. 2017 Dec 13;5:34. doi: 10.1186/s40364-017-0114-7 (PMC5729512; doi:10.1186/s40364-017-0114-7)

Supplemental Figure 1. Distribution of IL-10 and sTNF-RII in malaria infected versus uninfected women during pregnancy

Included are measurements of women (*n* = 80) included in the test set

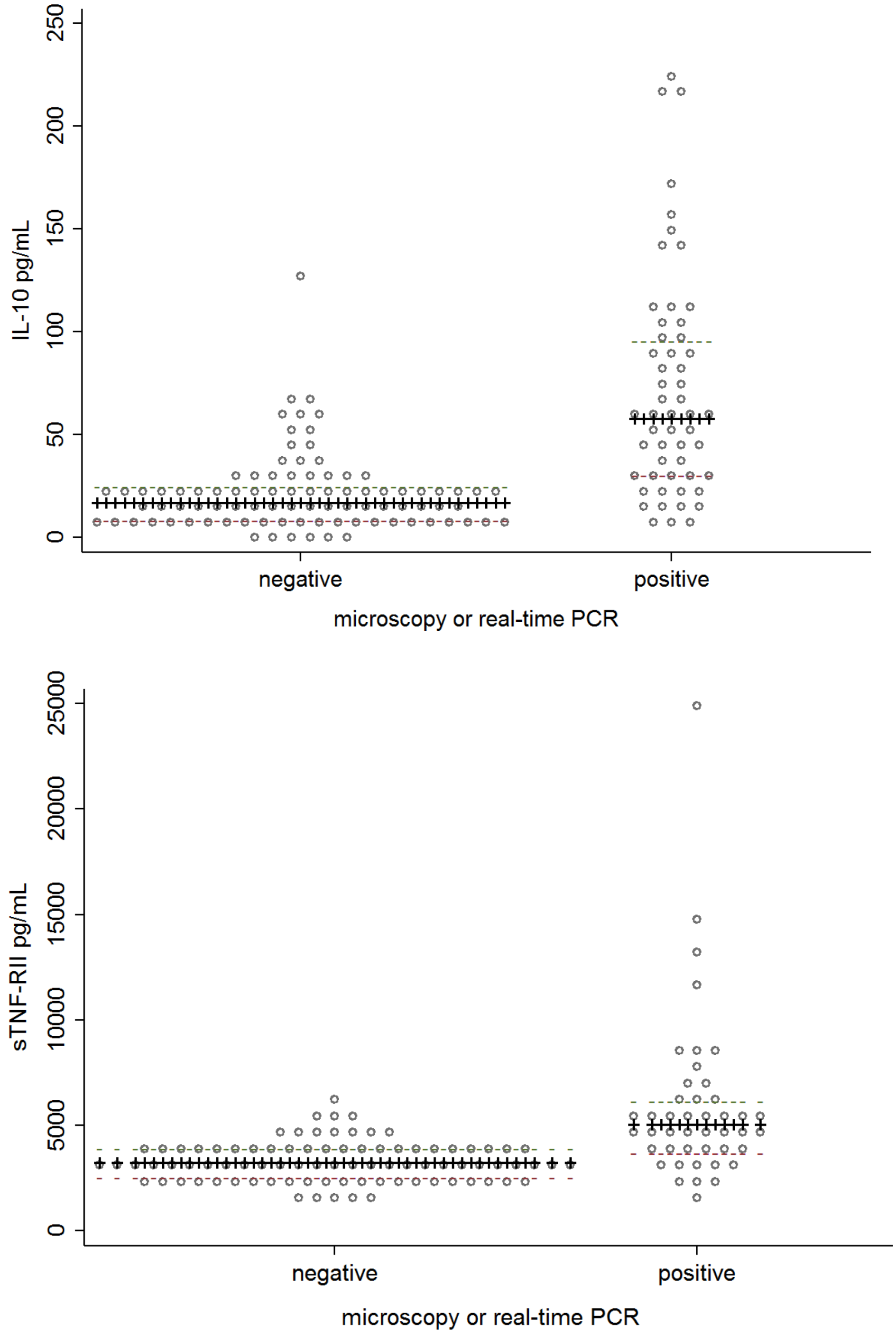

Supplement: Supplementary file 2 — Distribution of IL-10 and sTNF-RII in malaria infected versus uninfected women during pregnancy. (PDF 36 kb) [file 40364_2017_114_MOESM2_ESM.pdf]
